# Motor Abilities in Adolescents Born Preterm Are Associated With Microstructure of the Corpus Callosum

**DOI:** 10.3389/fneur.2019.00367

**Published:** 2019-04-16

**Authors:** Samuel Groeschel, Linda Holmström, Gemma Northam, J-Donald Tournier, Torsten Baldeweg, Beatrice Latal, Jon Caflisch, Brigitte Vollmer

**Affiliations:** ^1^Department of Child Neurology, Children's Hospital, University of Tübingen, Tübingen, Germany; ^2^Neuropaediatric Research Unit, Department of Women's and Children's Health, Karolinska Institutet Stockholm, Stockholm, Sweden; ^3^Developmental Neurosciences Programme, UCL Institute of Child Health, London, United Kingdom; ^4^Division of Imaging Sciences and Biomedical Engineering, Department of Biomedical Engineering, Centre for the Developing Brain, King's College London, London, United Kingdom; ^5^Child Development Center and Children's Research Centre, University Children's Hospital Zürich, Zurich, Switzerland; ^6^Clinical Neurosciences, Clinical and Experimental Sciences, Faculty of Medicine, University of Southampton, Southampton, United Kingdom

**Keywords:** preterm birth, brain injury, white matter microstructure, motor abilities, diffusion magnetic resonance imaging, tractography, corpus callosum

## Abstract

**Background:** Preterm birth is associated with increased risk of neuromotor impairment. Rates of major neuromotor impairment (cerebral palsy) have decreased; however, in a large proportion of those who do not develop cerebral palsy impaired neuromotor function is observed and this often has implications for everyday life. The aim of this study was to investigate motor performance in preterm born adolescents without cerebral palsy, and to examine associations with alterations of motor system pathway structure.

**Design/Methods:** Thirty-two adolescents (12 males) without cerebral palsy, born before 33 weeks of gestation (mean 27.4 weeks, SD 2.4; birth weight mean 1,084.5 g; SD 387.2), treated at a single tertiary unit, were assessed (median age 16 years; min 14, max 18). Timed performance and quality of movements were assessed with the Zürich Neuromotor Assessment. Neuroimaging included Diffusion Magnetic Resonance Imaging for tractography of the major motor tracts and measurement of fractional anisotropy as a measure of microstructure of the tracts along the major motor pathways. Separate analyses were conducted for areas with predominantly single and predominantly crossing fiber regions.

**Results:** Motor performance in both tasks assessing timed performance and quality of movements, was poorer than expected in the preterm group in relation to norm population. The strongest significant correlations were seen between performance in tasks assessing movement quality and fractional anisotropy in corpus callosum fibers connecting primary motor, primary somatosensory and premotor areas. In addition, timed motor performance was significantly related to fractional anisotropy in the cortico-spinal and thalamo-cortical to premotor area fibers, and the corpus callosum.

**Conclusions:** Impairments in motor abilities are present in preterm born adolescents without major neuromotor impairment and in the absence of focal brain injury. Altered microstructure of the corpus callosum microstructure appears a crucial factor, in particular for movement quality.

## Introduction

Very preterm birth (birth <32 weeks of gestation) is associated with high risk of impaired neurodevelopment. Rates of severe neuromotor impairment, i.e., Cerebral Palsy (CP), are decreasing, in particular in those preterm children born with moderately low and very low birth weight (1). However, in a substantial proportion of those born preterm who do not develop CP, delayed motor development, atypical neurological signs, and impaired neuromotor function is observed. This appears to occur across the preterm gestational age range and can persist throughout childhood, and there is now also some evidence that this continues into young adulthood (2–4). Often, motor difficulties co-occur with cognitive and/or behavioral difficulties (5, 6), and motor dysfunction is likely to contribute to the difficulties that are experienced at school and in social activities (7, 8), and can be associated with mental health (4). Overall, however, studies in adolescence and adulthood, are still sparse. In addition, most studies, with few exceptions (3, 9–12) have employed instruments such as the Movement Assessment Battery for Children or the Bruininks-Oseretsky Test of Motor Proficiency, that assess primarily motor development and motor skill level rather than specific aspects of motor abilities (13). Furthermore, many of the commonly used instruments may not be sufficiently sensitive to detect subtle, but nevertheless clinically relevant, difficulties in motor function.

It has been suggested that motor abilities may reflect internal neurological processes that underlie movement skills (14). Therefore, assessment of motor abilities appears an attractive approach for investigation of anatomical alterations in the motor system following preterm birth. The Zürich Neuromotor Assessment Battery (15–17) assesses motor abilities (including motor speed and quality of movements) in addition to movement skills (such as fine motor and balance skills), with good validity and reliability characteristics. It therefore provides a very suitable instrument to assess different aspects of motor abilities in at risk populations, in which only minor, but yet clinically relevant, motor deficits are expected.

There is a large body of literature which shows that brain growth and development is altered after preterm birth [see e.g., de Kieviet et al. (18), for review]. Studies have, for example, shown overall smaller total and regional white and gray matter brain volumes but also volume increases in some areas compared to term born individuals (19–21). A recent meta-analysis of diffusion magnetic resonance imaging (dMRI) studies (22) has identified consistent differences in fractional anisotropy (FA; often used as a measure of white matter, WM, microstructure) to term born individuals throughout childhood to young adulthood.

Little is known about how alterations in WM microstructure after preterm birth might be associated with specific deficits in motor abilities such as poor movement quality and impaired speed. Using dMRI-based fiber tracking in adolescents born preterm, we have previously described significant differences between preterm born and term born participants in measures of diffusion in a number of motor system pathways, namely cortico-spinal, thalamo-cortical, and transcallosal pathways, even in those where conventional MRI did not show overt signs of preterm brain injury (23).

In the present study, we investigated (1) whether in those preterm participants without CP, specific motor abilities that are relevant for daily activities are impaired, and (2) whether this is associated with the previously identified alterations of microstructure, indicated by FA as a measure of white matter microstructure, along motor pathways.

## Materials and Methods

### Participants

The participants were 32 adolescents born <33 weeks of gestation, treated at University College London Hospitals, London, UK, a level III unit, between 1989 and 1994. Mean gestational age at birth was 27.4 weeks (SD 2.4; min 23, max 31 weeks), birth weight mean 1,084.5 g (SD 387.2; min 591; max 2,243). Median age at assessment was 16 years (min 14, max 18); there were 12 males and 20 females. This sample is a subset of the sample that was investigated in the above mentioned previous study (23). Only participants without CP, who completed the ZNA, and for whom good quality dMRI data were available, were included. This subset did not differ significantly from the overall sample in relevant demographic or perinatal variables. On radiological assessment 14 participants had normal MRI; periventricular signal abnormalities on T2-weighted images only were seen in 3/32, and WM reduction/ ventricular dilatation (judged by visual inspection) in 15/32 participants, of which 13 were mild/moderate (≤50% of the periventricular WM bulk reduced, and two severe (>50%) WM reduction. Abnormalities were bilateral in 11 participants. One participant received physiotherapy at the time of this study, five had had physiotherapy at some point in the past; 10 had been provided with glasses, and 2/10 had visual impairment that was not fully corrected by glasses; for none of these two participants difficulties with the ZNA tasks were observed. All except 2 (1 at special school, 1 at mainstream school with some extra help) attended mainstream school without extra help. Mean Full Scale IQ was 94.5 (SD 14.9; min 65, max 120), Verbal IQ was 93.3 (SD 13.1; min 70, max 115), Performance IQ was 96.8 (SD 15.9; min 67, max 129), measured with the Wechsler Abbreviated Scale of Intelligence, at time of this study. All participants were able to understand the instructions given for the ZNA.

The study was approved by the local ethics committee (Institute of Child Health and Great Ormond Street Hospital; REC reference 04/Q0508/86) and written informed consent was obtained from all participants and their parents.

### Procedure

Neuromotor assessment and neuroimaging were performed on the same day for all participants.

### Assessment of Neuromotor Function

Neuromotor function was assessed with the Zürich Neuromotor Assessment Battery (15–17, 24, 25). The ZNA is a standardized testing procedure which consists of a number of motor tasks for assessment of timed performance (speed of movements) and movement quality (associated movements); it is a reliable (26) and validated (10, 27) measure, covering the age range 5–18 years.

The assessment is videotaped and scored off-line. *Timed performance* is determined by assessing speed of movements; this is done to an accuracy of one tenth of a second, with exact beginning of time measurement and the number of movements to be measured having been established for each individual motor task. The measures include repetitive, alternating, and sequential tasks for fingers, hands, and feet; and also a pegboard task, a static balance task, and two dynamic balance tasks (side- and forward jumping). *Movement quality* is assessed by scoring of associated movements. Associated movements are defined as involuntary movements in parts of the body which are not actively involved in the task. The less frequent and the less marked the associated movements are, the higher the movement quality. Associated movements are assessed for frequency and degree. While the active extremity carries out the required number of movements, the frequency of associated movements is noted in tenths of the number of active movements (score ranges from 0 to 10). The degree of associated movements is judged based on the maximum possible movement range for the observed associated movement according to a four-point scale (score ranges from 0 to 3).

Results are expressed as “components”: (1) *pure motor component*, which consists of timed performance of all repetitive, alternating, and sequential tasks; (2) *adaptive fine motor component*, which consists of the timed performance of the pegboard; (3) *adaptive gross motor component*, which combines dynamic balance tasks, and (4) *static balance component*, which includes the static balance task; (5) *associated movement component*, which consists of all results from the associated movement tasks and stress gaits.

The ZNA also allows calculation of so called “differential components,” which are designed to capture differences in performance between left and right limbs, and upper and lower extremities. In our study, only the block components pure motor, adaptive fine motor, adaptive gross motor, static balance, and associated movements were used.

Standard deviation scores (SDS) are calculated from age- and sex-adjusted normative values (16, 17), i.e., performance at a specific age is expressed as a number of SD (*z*-scores) above or below the average performance of children/adolescents of the same age and sex. For the analyses carried out in the here described study, it is important to point out that negative values of the SDS scores reflect better performance.

Intra-rater reliability for the motor components in “timed performance” measured by intraclass correlation is 0.85–1, inter-rater reliability 0.83–0.98, and test-retest reliability 0.57–0.91; for the components in “contralateral associated movements” intra-rater reliability is 0.73–0.85, inter-rater reliability 0.52–0.79, and test-retest reliability from 0.40–0.66 (26).

### MR Image Acquisition

Images were acquired on a 1.5 Tesla Siemens Avanto Scanner (Erlangen, Germany). The protocol consisted of conventional T2-weighted images (axial multi-slice sequence; repetition time [TR] = 4,920 ms, echo time [TE] = 101 ms, field of view = 220 mm, slice thickness = 4 mm, slices = 25, matrix size = 384 × 384); 3-D T1-weighted data sets [fast low angle shot (3D-FLASH) sequence, TR = 11 ms, TE = 4.94 ms, flip angle = 15°, field of view = 256 mm, matrix size = 256 × 256, voxel size = 1 × 1 × 1 mm], and a 3D T2-weighted fluid attenuated inversion recovery sequence (TR = 6,000 ms, TE = 353 ms, TI = 2200 ms, flip angle = 150°, field of view = 256 mm, matrix = 256 × 256, voxel size = 1 × 1 × 1 mm), The diffusion-weighted (DW) sequence consisted of a high angular resolution twice-refocused echo planar imaging (EPI) sequence (*b* = 3000 s/mm^2^, 60 DW directions, TE/TR = 128/7,700 ms, 112 × 112 matrix, FOV = 235 × 235 mm, slice thickness = 3 mm, voxel size = 2.1 × 2.1 × 3 mm, 37 contiguous slices).

### Diffusion MR Data Processing and Analysis

**Figure 1:**
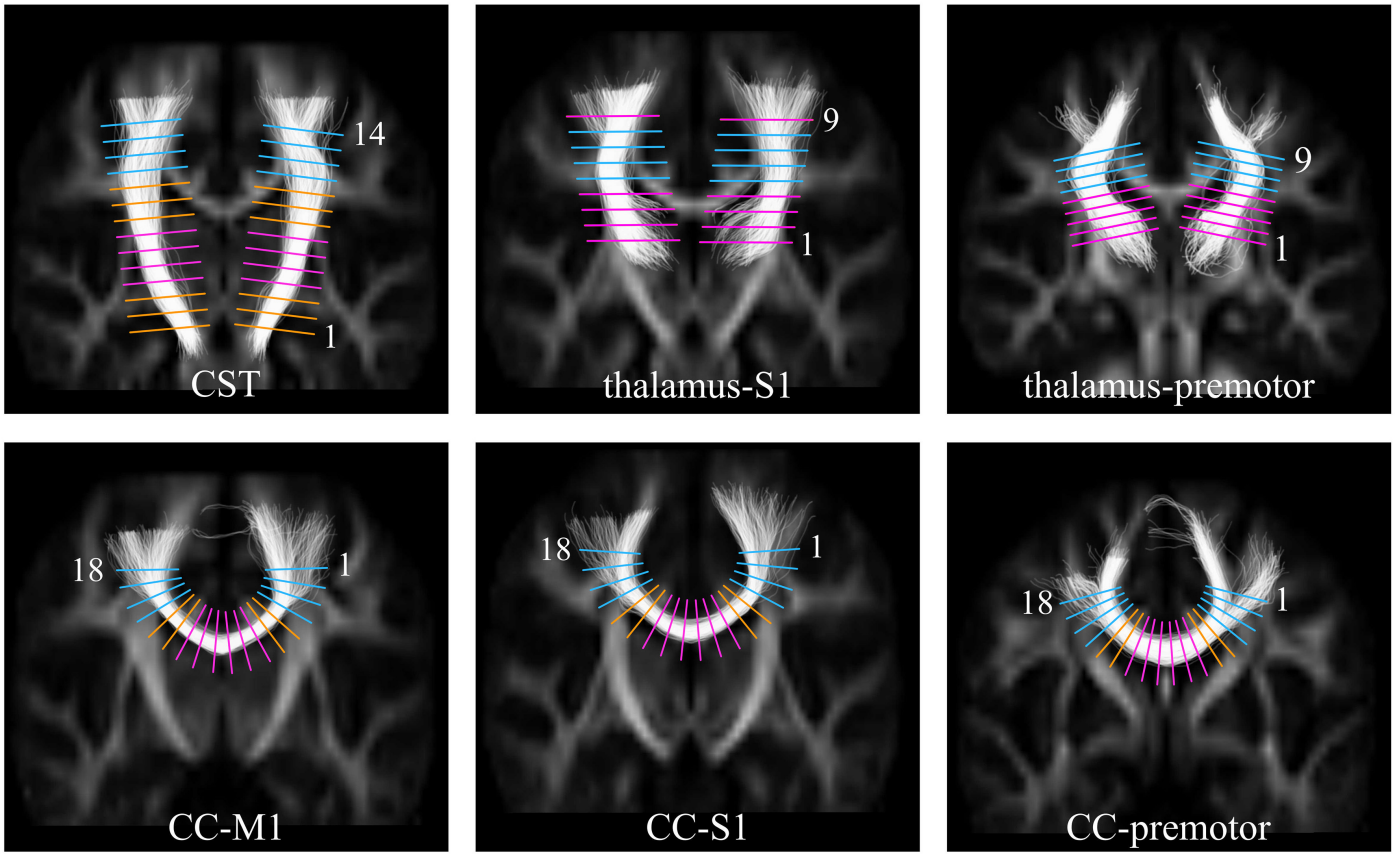
Images showing the positions in template space at which FA was sampled along each of the white matter tracts. **Top Row:** the cortico-spinal tract (CST), the thalamus to S1, the thalamus to premotor cortex; **Bottom Row:** the callosal fibers between M1 (CC-M1), S1 (CC-S1), and premotor (CC-premotor) cortices. The sample levels categorized as being through predominantly single fiber regions are shown in pink, and through crossing fiber regions in the centrum semiovale shown in blue. For the orange levels no prior hypotheses have been formulated [This figure has previously been published in Groeschel et al. (23), and is used here in a slightly modified version; permission to use this figure has been obtained].

**Figure 2:**
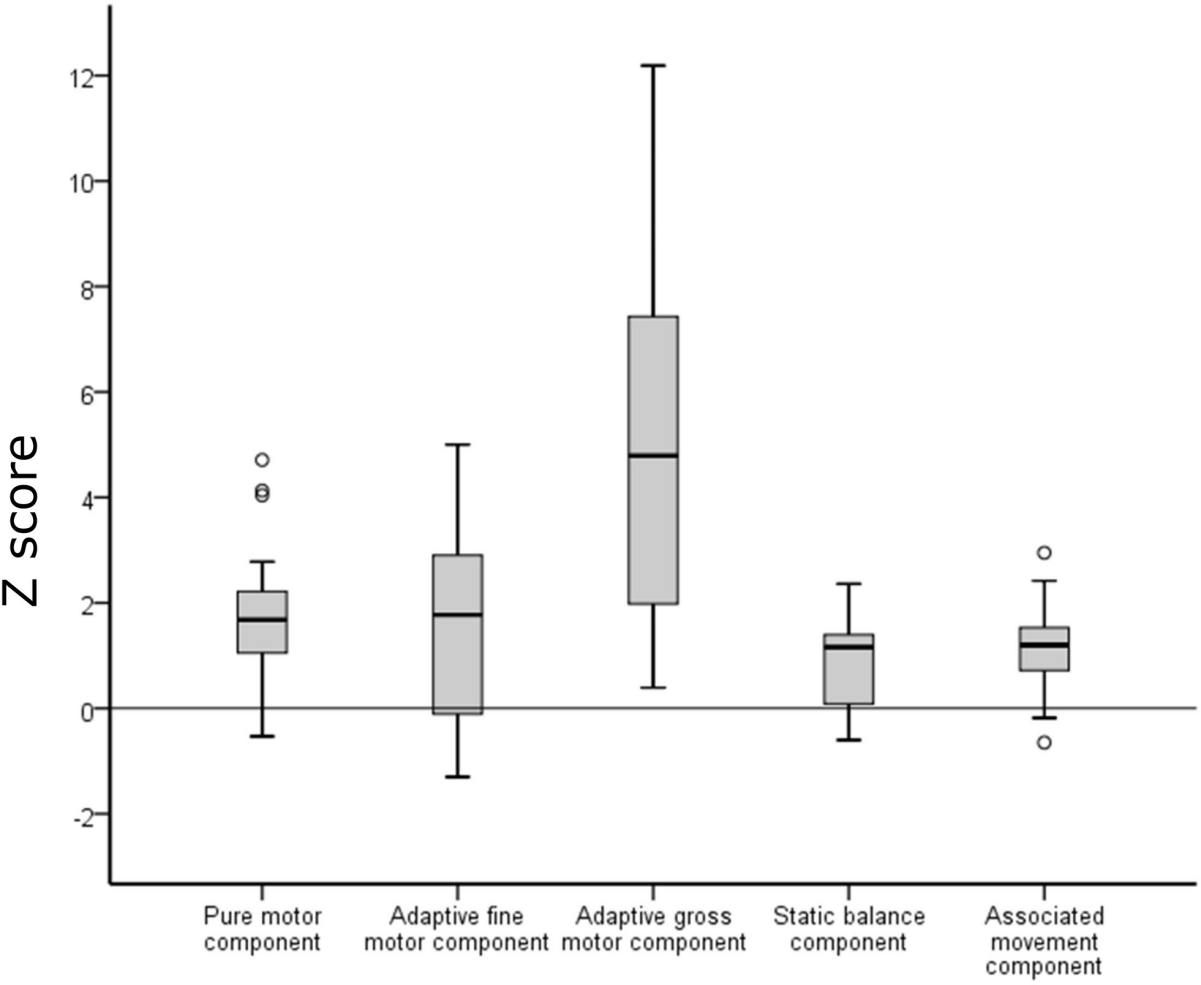
Performance on the Zürich Neuromotor Assessment (ZNA) in the preterm group (box plots) in relation to the norm median (black line). Negative *z*-scores indicate better performance and positive z-scores indicate poorer performance compared to the reference data of the normative population. Empty circles indicate outliers.

Processing and analysis of the diffusion MR data have been described in detail in Groeschel et al. (23). In brief, all diffusion images were visually inspected for motion and other artifacts (e.g., eddy current artifacts) and those with artifacts were excluded. Following calculation of diffusion tensor images, FA-images were spatially normalized to a study specific FA-template; color-coded major eigenvector templates were created for visualization purposes; following this, non-linear large deformation mapping was performed. The FA- and eigenvector templates were used to define seed and target regions of interest (ROI) for tractography [see Figure 2 (23)]. Target ROIs included bilateral M1, S1, and premotor areas, while seed regions consisted of the thalamus or cerebral peduncle (or the contralateral M1/S1/premotor areas for callosal pathways) (23). Probabilistic fiber tracking was based on the fiber orientations estimated via constrained spherical deconvolution (28), combined with a probabilistic streamlines algorithm as implemented in MRtrix (29). This approach allows reliable fiber tracking through regions with crossing fibers avoiding the known limitations related to DTI-based tractography (30, 31). The ROIs defined in template space and warped to each individual's native space were used as seed and target regions for the tracking and the delineated tracts included the cortico-spinal tract (cerebral peduncle –M1), thalamo-cortical connections to primary sensory cortex (thalamus -S1), and premotor areas (thalamus -premotor), as well as transcallosal fibers connecting bilateral M1, S1, and premotor areas separately. Diffusion parameters were measured along the delineated tracts at equally spaced planes as visualized in Figure 1 and described in more detail previously (23), which were defined in template space for each tract and then warped into each individual dataset's native space. For the current study we used FA in the analyses that investigated correlations between motor performance scores and neuroimaging.

We have previously shown (23) in this cohort of preterm adolescents that white matter microstructure in motor pathways is altered and that diffusion parameters are affected differently depending on the underlying fiber architecture. Disruption of WM microstructure in a predominantly single fiber region (e.g., internal capsule) with resulting higher radial diffusivity would lead to lower FA, whereas selective disruption of one fiber population in a region with a high proportion of crossing fibers (e.g., centrum semiovale) may lead to higher FA. Therefore, for the analyses investigating associations between motor performance and FA, separate analyses were performed for predominantly single fiber and predominantly crossing fiber regions. Figure 1 illustrates the levels along the tracts corresponding to these respective areas. Predominantly single fiber regions were defined based on anatomical knowledge for the cortico-spinal tract at the level of the internal capsule (levels 4–7), and for the callosal pathways in the midsagittal area of the corpus callosum (levels 7–12). Predominantly crossing fiber regions were defined in the centrum semiovale, corresponding to levels 11–14 for the cortico-spinal tract; levels 5–8 for the thalamo-cortical pathway to S1; levels 6–9 for the anterior thalamic radiation, and levels 1–4 and 15–18 for all three callosal pathways.

### Statistical Analysis

Zero-order correlations between the different components of the ZNA were calculated using Pearson's *r*. Partial correlations were calculated to control for possible age effects in the analyses. For the analyses examining correlations between motor measures and FA, for each tract under investigation, all single fiber regions were combined and similarly all crossing fiber regions were combined. Averages were calculated between the right and left cortico spinal tracts and thalamo-cortical tracts, respectively. This was done as we have shown previously that both sides differ in mean in the same manner between preterms and controls, underlining that the long-term effect of preterm brain injury appears to affect the microstructure of the brain white matter in a similar way bilaterally (23). Since it would be reasonable to expect that motor performance is different between those with and without macroscopic periventricular MRI abnormalities we performed a comparison of motor performance between the group with and those without macroscopic periventricular WM abnormalities by Mann-Whitney-U test to rule out that a possible difference might affect our correlation analyses. Tests were two-tailed and *p* < 0.05 as cut-off level for significance was chosen. Analyses were performed in SPSS 22.

The presented *p*-values were not corrected for multiple comparisons using Bonferroni correction, as it falsely assumes all tests to be independent, which they were not, (since) partly overlapping in location. Therefore, the *p*-values should be regarded as uncorrected.

## Results

### Performance on the ZNA

Results of the neuromotor tests are presented in Figure 2. Zero-order correlations between ZNA components are presented in Table 1. There were significant moderate-high positive correlations (see Table 1) between the pure motor component and all other components, and between the adaptive fine motor task and adaptive gross motor task, respectively.

**Table 1:** Zero-order correlations between the components of the Zürich neuromotor assessment (ZNM).

	**Adaptive fine motor component**	**Adaptive gross motor component**	**Static balance component**	**Associated movement component**
	***R***	***r***	***r***	***r***
Pure motor component	0.550^**^	0.716^**^	0.417^*^	0.396^*^
Adaptive fine motor component		0.504^**^	0.260	0.281
Adaptive gross motor component			0.191	0.345
Static balance component				0.331

* *Correlation is significant at the 0.05 level (2-tailed)*.

** *Correlation is significant at the 0.01 level (2-tailed)*.

*r = correlation coefficient*.

Results of our preterm sample are presented in relation to performance of the normal population of the ZNA. For all tasks, performance in the preterm group was poorer than in the normal population (Figure 2). However, differences varied between the different components. For the pure motor tasks, 43.7% of the preterm group performed below 2 SD from the mean expected at this age, for adaptive motor tasks 37.5%, static balance 3.2%, and associated movements 12.5%. The difference was most pronounced for the tasks of dynamic balance (double jumps sideways and of forward jumps within two lines) where 74% performed below 2 SD; however, for this component, there was a large variability in performance which was mainly due to poor participant compliance in this specific task.

### Relationships Between Motor Performance and Macroscopic Brain Abnormalities

Group comparison for differences in task performance between those with and those without periventricular WM abnormalities showed only weak evidence for a difference between these two groups for performance in associated movement tasks (*p* = 0.054), and pure motor tasks (*p* = 0.059).

### Relationships Between Motor Performance and FA in Motor Pathways

**Figure 3:**
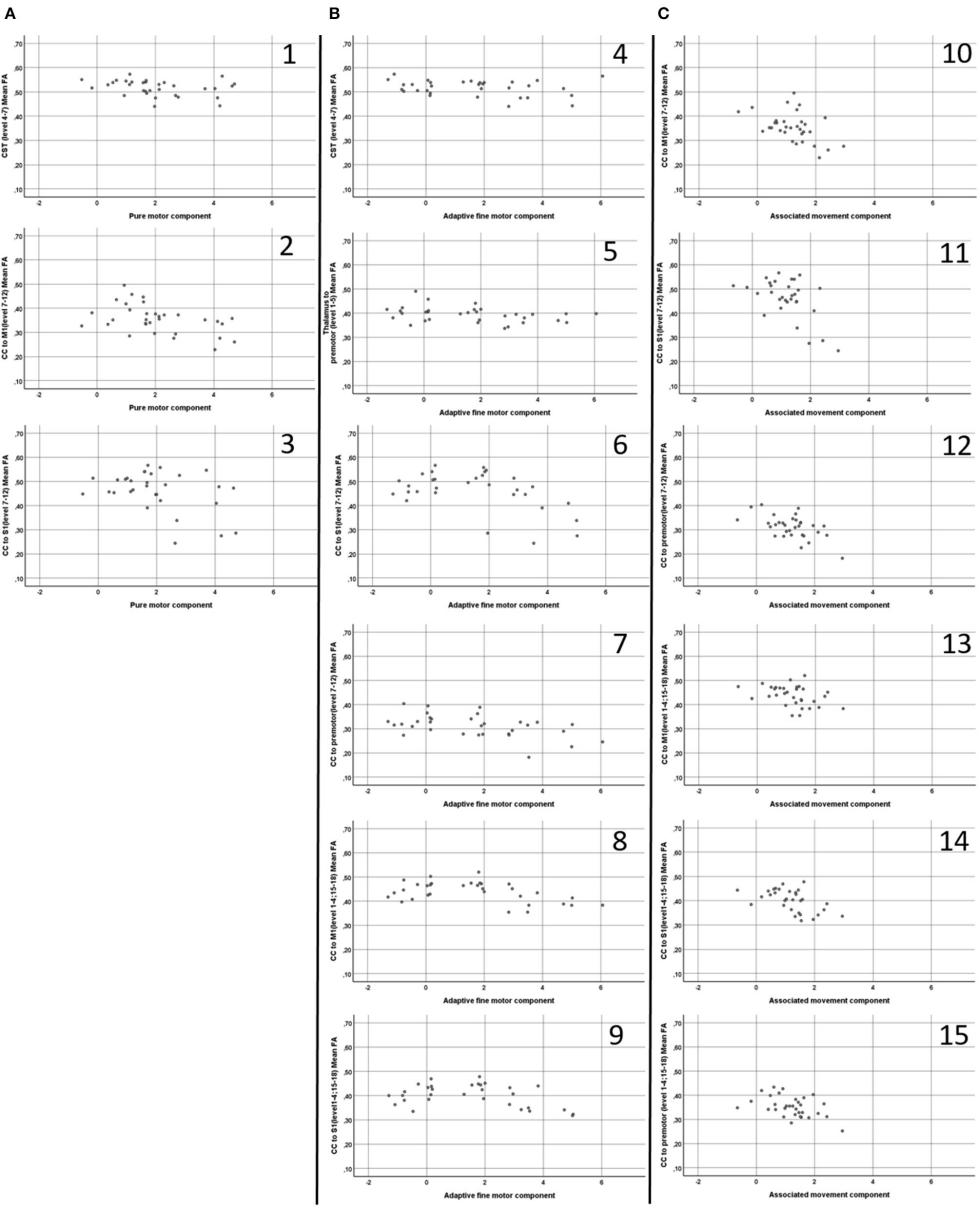
Scatterplots **(A1–3,B 4–9,C10–15)** of direct relationships (i.e. not partial controlled for age) between mean Fractional Anisotropy (FA) (Y axis) and performance on Zürich Neuromotor Assessment components (X axis), displayed for significant correlations. CST, Cortico-spinal tract; CC, Corpus Callosum; M1, Primary motor cortex 1; S1, Sensory cortex. A1; CST (level 4-7) mean FA and Pure Motor component; A2, CC to M1 (level 7-12) mean FA and Pure Motor component; A3, CC to S1 (level 7–12) mean FA and Pure Motor component; B4, CST (level 4–7) mean FA and Adaptive Fine Motor component; B5, Thalamus to Premotor (level 1–5) mean FA Adaptive Fine Motor component; B6, CC to S1 (level 7–12) mean FA and Adaptive Fine Motor component; B7, CC to Premotor (level 7–12) mean FA and Adaptive Fine Motor component; B8, CC to M1 (level 1–4: 15–18) mean FA and Adaptive Fine Motor component; B9, CC to S1 (level 1–4: 15–18) mean FA and Adaptive Fine Motor component; C10, CC to M1 (level 7–12) mean FA and Associated Movement component; C11, CC to S1 (level 7–12) mean FA and Associated Movement component; C12, CC to Premotor (level 7–12) mean FA and Associated Movement component; C13, CC to S1 (level 1–4: 15–18) mean FA and Associated Movement component; C14, CC to M1 (level 1–4: 15–18) mean FA and Associated Movement component; C15, CC to Pre-Motor (level 1–4: 15–18) mean FA and Associated Movement component.

Table 2 details the findings from the partial correlation analyses examining associations between motor performance and FA. Figure 3 shows scatterplots of direct relationships between mean FA and performance on Zürich Neuromotor Assessment components for significant correlations. For both areas with predominantly single fiber populations and areas with predominantly crossing fiber populations, the most consistent and strongest correlations were seen between FA and quality of movements (associated movements). Measures of associated movements were strongly related to FA in all three portions of the corpus callosum, i.e., in fibers connecting the M1 areas (*r* = −0.43, *p* = 0.018), the S1 areas (*r* = −0.506, *p* = 0.004), and premotor areas (*r* = −0.531, *p* = 0.003). In addition, adaptive fine motor measures were related to FA in the CST (*r* = −0.435, *p* = 0.016) and thalamo-cortical to premotor area fibers (*r* = −0.411, *p* = 0.024) in predominantly single fiber populations only; to FA in the CC [fibers linking S1 and premotor areas in predominantly single fibers population regions (*r* = −0.438, *p* = 0.016), and M1 (*r* = −0.41, *p* = 0.024) and S1 (*r* = −0.389, *p* = 0.034) areas in predominantly crossing fiber regions]. Finally, performance on pure motor tasks was related to FA in the CST (*r* = −0.392, *p* = 0.032) and CC (M1 to M1, *r* = −0.479, *p* = 0.007; and S1 to S1, *r* = −0.41, *p* = 0.024) in regions with predominantly single fiber populations only.

**Table 2:** Partial correlations between performance on the Zürich Neuromotor Assessment (ZNM) components and Fractional Anisotropy (FA).

**Partial correlations controlled for age**		**Pure motor component**		**Adaptive fine motor component**		**Static balance component**		**Associated movement component**	
		***r***	**95% CI lower/upper**	***r***	**95% CI lower/upper**	***r***	**95% CI lower/upper**	***r***	**95% CI lower/upper**
Predominantly single fiber population	CST (level 4–7)	−0.392^*^	**−0.680/−0.114**	−0.435^*^	**−0.654/−0.147**	−0.340	−0.778/0.100	−0.086	−0.417/0.300
	Thalamus to S1 (level 1–4; 9)	−0.158	−0.470/0.105	−0.154	−0.462/0.171	0.039	−0.356/0.376	−0.231	−0.612/0.061
	Thalamus to premotor (level 1–5)	−0.299	−0.559/−0.041	−0.411^*^	**−0.625/0.106**	0.011	−0.460/0.369	−0.309	−0.019/0.106
	CC to M1 (level 7–12)	−0.479^**^	**−0.716/−0.201**	−0.282	−0.560/0.107	−0.191	−0.511/0.089	−0.430^*^	**−0.693/−0.121**
	CC to S1 (level 7–12)	−0.410^*^	**−0.638/−0.115**	−0.438^*^	**−0.659/−0.099**	−0.162	−0.496/0.249	−0.506^**^	**−0.741/–0.157**
	CC to premotor (level 7–12)	−0.141	−0.392/0.156	−0.400^*^	**−0.672/−0.025**	−0.209	−0.483/0.132	−0.531^**^	**−0.758/−0.178**
Predominantly crossing fiber population	CST (level 11–14)	−0.179	−0.567/0.183	−0.320	−588/−0.045	−0.176	−0.478/0.148	−0.205	−64/0.070
	thalamus to S1 (level 5–8)	−0.038	−0.445/0.326	−0.281	−0.520/0.008	0.016	−0.333/0.311	−0.148	−0.404/0.091
	thalamus to premotor (level 6–9)	0.112	−0.152/0.384	−0.003	−0.321/0.324	0.092	−0.345/0.333	−0.155	−0.388/0.061
	CC to M1 (level 1–4;15–18)	−0.226	−0.015/0.632	−0.410^*^	**−0.660/−041**	0.012	−0.534/0.320	−0.370^*^	**−0.644/−0.098**
	CC to S1 (level 1–4;15–18)	−0.256	−0.533/0.063	−0.389^*^	**−0.670/0.071**	−0.130	−0.522/0.249	−0.533^**^	**−0.713/−0.291**
	CC to premotor (level 1–4;15–18)	−0.074	−0.347/0.230	−0.283	−0.596/0.094	−0.077	−0.534/0.320	−0.419^*^	**−0.710/−0.074**

* *Correlation is significant at the 0.05 level (2-tailed)*.

** *Correlation is significant at the 0.01 level (2-tailed)*.

*r = correlation coefficient*.

*CI, confidence interval*.

*Significant results are in bold*.

## Discussion

The present study investigated specific aspects of motor abilities, namely timed performance and quality of movements, and associations with microstructure (indicated by FA) of motor pathways in adolescents born very preterm without CP. The primary finding of this study is the consistent significant relationship between FA in different portions of the callosal fibers and performance on the ZNM components assessing timed performance and quality of movements.

The preterm group performed poorer than expected in relation to the reference data of the ZNA, and there was a larger variation in performance between the components in relation to the norm population. The significant positive correlations between the pure motor component and all other components indicate that individual performance level was consistent across components.

When examining associations between motor performance and FA in the white matter tracts of interest, the strongest correlations were seen between the associated movement component and FA in the CC, although the proportion of preterms with poor performance was relatively low for this component. It can be argued that this finding is a result of the complexity of the different components, where performance of the more complex tasks requires a more extensive involvement of the motor network in both hemispheres. This argument may be further supported by the significant relationship between FA in several of the other structures (CST, thalamo-cortical to premotor area fibers and CC fibers linking S1 and premotor areas) and the adaptive fine motor component, since performance in this task is also likely to be highly dependent on an intact and efficient network.

Injury to the motor system remains overall the most common injury in the context of preterm birth (32, 33) and, even in the absence of CP, can have negative impact on fine motor abilities such as reduced motor speed (3, 9), quality of movements (14, 15), as well as motor skills such as balance, manual dexterity (33), and visuo-motor skills (12, 34). Several recent papers have used advanced neuroimaging techniques to investigate and describe associations between motor impairments and alterations in motor tracts, as described by FA, in individuals with focal brain lesions resulting in CP [see, for example (35–38)]. There is, however, a high proportion of preterm born individuals who do not develop CP, and, to date, the literature on neural correlates of subtle motor impairment in preterm adolescents overall is sparse. However, the presence of specific types of mild motor deficits that mainly affect the quality of movements rather than severely impacting on function have been reported earlier in the literature. For instance, children born very preterm have been shown to have problems with movement organization, with slower and less smooth movement trajectories compared to term born peers (11). Furthermore, findings from a previous study on very low birthweight children using the ZNA, show poorer abilities in timed motor performance and movement quality in relation to norms (11).

The neuromotor difficulties in our sample should be considered mild, and are mainly related to subtle problems with quality, speed and coordination of movements in complex tasks. Nevertheless, performance was correlated with white matter microstructure measures in several of the motor tracts, primarily in the single fiber populations of the CC. These findings are in line with a previous study showing relationships between FA in several WM structures, including the CC, and motor skills in very low birthweight adolescents (39). Hollund et al. (40), investigated a subgroup of the cohort that formed the basis of the study by Skranes et al. (39), at age 23 years, and found subtle differences between the very-low-birth weight group and term born controls in timed performance of fine motor tasks and, to a lesser degree, some gross motor tasks, and this was associated with FA alterations along the CST and the CC, although they found lower FA only in crossing but not in single fiber regions of the CC in their cohort.

Our findings suggest that we should consider the motor deficits seen in preterm born individuals as a result of alterations in not just one tract but in a complex network. The intact structure of the CC has been suggested a necessary component in the network responsible for both information processing and transmission in bimanual task performance (41). It has also been suggested that efficient motor performance relies on an intrinsic balance of excitatory and inhibitory couplings, connecting nodes of the motor system within and across hemispheres, and the callosal fibers play an important role in this network (42–44). This has also been demonstrated in a recent study of children after neonatal stroke, where transcallosal motor fibers were associated with motor function of both hands (45). The connections are somatotopically organized (46) and the quality of these interhemispheric connections are strongly influenced by sex, age and motor training in addition to size of the CC (42, 47). Moreover, findings combining measures of FA in callosal fibers with paired-pulse transcranial magnetic stimulation as a measure of interhemispheric inhibition provide evidence that FA in the CC is closely linked to functional connectivity (42). The mediating function of the CC has also been implied in the context of motor “overflow” (48), which refers to involuntary movements that accompany voluntary movements during development, in the elderly and in some individuals with neurological dysfunction (48, 49).

Our results point to CC microstructure as a possibly crucial factor with regards to degree of motor impairment in complex motor tasks with high demands on coordination and timing of movements in individuals born very preterm. From a clinical point of view, it would be of great interest to examine in prospective studies whether FA in the CC measured in infancy might serve as an early marker for future motor development.

The advantage of the present study lies in the use of advanced neuroimaging techniques in combination with a norm referenced motor assessment that investigates both motor abilities and skills, and which is sensitive to the specific but minor motor problems expected in this population. In addition, we have separately analyzed single and crossing fiber areas, which should increase the sensitivity of our analyses further. One can only speculate whether or not the specific motor problems seen in our sample are a result of a developmental delay that may improve over time, or signs of permanent deficits. Motor abilities and skills that are tested with the ZNA all have developmental trajectories that are expected to level off in the teenage years, with some tasks showing large inter-individual variation, for example, timed performance tasks (24, 25). The fact that the participants in the present study are at adolescent age would support the view that the observed motor deficits are permanent rather than simply a developmental delay in which catch-up can be expected.

Some limitations should be taken into consideration when interpreting our findings. The use of cross-sectional data does not permit any investigations of causality and the relationships seen between FA and motor abilities should be interpreted with this in mind. While our results need to be confirmed by other cohorts with larger sample sizes, we remain confident that our results from this relatively small sample are valid and provides additional guard against trivial effects (50). It should also be noted, that the p-values are reported as uncorrected for multiple comparisons. In fact, it is difficult to use the right form of correction as Bonferroni correction assumes that each test is independent, which they are not, as several tracts are used in the same patient; in addition, tracts overlap heavily in location. We aimed to minimize the problem of multiple comparisons by using a very specific prior hypothesis, combining several measurement levels to certain regions (predominantly single vs. crossing fibers) and used FA as single DTI metric. In addition, the risk of partial volume effects as a result of enlarged ventricles/thinning of the CC influencing FA cannot be ignored. Furthermore, FA metrics are known to be problematic in crossing fiber regions, however, still valid to use in predominantly single fiber regions (31). While in this work we adapted our methodology to focus on these regions in certain motor pathways, future work investigating whole-brain microstructural changes might use non-DTI metrics, such as fiber density [e.g., (51)], to overcome this limitation. We have compared motor test performance of the preterm participants with the published normative ZNA data, which is common practice, and will identify atypical neuromotor function reliably. However, it would be of interest in further work to include a contemporaneous control group of term born individuals.

## Conclusions

Impairment of motor abilities is present at adolescent age in preterm individuals without CP. This is related to altered microstructure in various motor tracts, and our findings suggest that altered microstructure of the CC is a crucial factor associated with impaired timed performance and quality of movements in the context of preterm birth.

## Ethics Statement

This study was approved by the local ethics committee (Institute of Child Health and Great Ormond Street Hospital; REC reference 04/Q0508/86) and written informed consent was obtained from all participants and their parents in accordance with the Declaration of Helsinki.

## Author Contributions

LH, BV, SG, J-DT, BL, JC, GN, and TB contributed conception and design of the study. BV, J-DT, and GN contributed to data collection. LH and GN organized the database. BV and LH performed the statistical analysis. SG and J-DT performed the imaging analysis. LH and BV wrote the first draft of the manuscript. TB, J-DT, SG, BL, and JC wrote sections of the manuscript. All authors contributed to manuscript revision, read, and approved the submitted version.

### Conflict of Interest Statement

The authors declare that the research was conducted in the absence of any commercial or financial relationships that could be construed as a potential conflict of interest.

## Abbreviations

CC: corpus callosum
CP: cerebral palsy
CST: cortico-spinal tract
DTI: diffusion tensor imaging
FA: fractional anisotropy
M1: primary motor cortex
MD: mean diffusivity
ROI: region of interest
S1: sensory motor cortex
SD: standard deviation
SDS: standard deviation scores
WM: white matter
ZNA: Zürich Neuromotor Assessment.

## References

[1] Sellier E Platt MJ Andersen GL Krageloh-Mann I De La Cruz J Cans C. Decreasing prevalence in cerebral palsy: a multi-site European population-based study, 1980 to 2003. Dev Med Child Neurol. (2016) 58:85–92. 10.1111/dmcn.12865 26330098

[2] Pitcher JB Schneider LA Drysdale JL Ridding MC Owens JA. Motor system development of the preterm and low birthweight infant. Clin Perinatol. (2011) 38:605–25. 10.1016/j.clp.2011.08.010 22107893

[3] Husby IM Skranes J Olsen A Brubakk AM Evensen KA. Motor skills at 23 years of age in young adults born preterm with very low birth weight. Early Hum Dev. (2013) 89:747–54. 10.1016/j.earlhumdev.2013.05.009 23810435

[4] Husby IM Stray KM Olsen A Lydersen S Indredavik MS Brubakk AM Long-term follow-up of mental health, health-related quality of life and associations with motor skills in young adults born preterm with very low birth weight. Health Qual Life Outcomes. (2016) 7:14:56. 10.1186/s12955-016-0458-y PMC4823914 27052007

[5] Schneider LA Burns NR Giles LC Nettelbeck TJ Hudson IL Ridding MC. The influence of motor function on processing speed in preterm and term-born children. Child Neuropsychol. (2017) 23:300–15. 10.1080/09297049.2015.1102215 26507931

[6] Van Hus JW Potharst ES Jeukens-Visser M Kok JH Van Wassenaer-Leemhuis AG. Motor impairment in very preterm-born children: links with other developmental deficits at 5 years of age. Dev Med Child Neurol. (2014) 56:587–94. 10.1111/dmcn.12295 24926490

[7] Dahan-Oliel N Mazer B Riley P Maltais DB Nadeau L Majnemer A. Participation and enjoyment of leisure activities in adolescents born at < / = 29 week gestation. Early Hum Dev. (2014) 90:307–14. 10.1016/j.earlhumdev.2014.02.010 24661444

[8] Wocadlo C Rieger I. Motor impairment and low achievement in very preterm children at eight years of age. Early Hum Dev. (2008) 84:769–76. 10.1016/j.earlhumdev.2008.06.001 18639396

[9] Pitcher JB Schneider LA Burns NR Drysdale JL Higgins RD Ridding MC. Reduced corticomotor excitability and motor skills development in children born preterm. J Physiol. (2012) 590:5827–44. 10.1113/jphysiol.2012.239269 22966161 PMC3528994

[10] Schmidhauser J Caflisch J Rousson V Bucher HU Largo RH Latal B. Impaired motor performance and movement quality in very-low-birthweight children at 6 years of age. Dev Med Child Neurol. (2006) 48:718–22. 10.1111/j.1469-8749.2006.tb01355.x 16904016

[11] Johansson AM Domellof E Ronnqvist L. Long-term influences of a preterm birth on movement organization and side specialization in children at 4–8 years of age. Dev Psychobiol. (2014) 56:1263–77. 10.1002/dev.21206 24523104

[12] Jongmans M Mercuri E de Vries L Dubowitz L Henderson SE. Minor neurological signs and perceptual-motor difficulties in prematurely born children. Arch Dis Childh Fetal Neonatal Ed. (1997) 76:F9–14. 9059179 10.1136/fn.76.1.f9 PMC1720611

[13] Kakebeeke TH Egloff K Caflisch J Chaouch A Rousson V Largo RH. Similarities and dissimilarities between the movement ABC-2 and the Zurich neuromotor assessment in children with suspected developmental coordination disorder. Res Dev Disabil. (2014) 35:3148–55. 10.1016/j.ridd.2014.07.062 25151604

[14] Burton AW Rodgerson RW New perspectives on the assessment of movement skills and motor abilities. Adapt Phys Activity Q. (2001) 18:347–65. 10.1123/apaq.18.4.347

[15] Largo RH Fischer JE Rousson V. Neuromotor development from kindergarten age to adolescence: developmental course and variability. Swiss Med Wkly. (2003) 133:193–9. 10.5167/uzh-34720 12811675

[16] Largo RH Caflisch JA Hug F Muggli K Molnar AA Molinari L Neuromotor development from 5 to 18 years. Part 1: timed performance. Dev Med Child Neurol. (2001) 43:436–43. 10.1111/j.1469-8749.2001.tb00739.x 11463173

[17] Largo RH Caflisch JA Hug F Muggli K Molnar AA Molinari L Neuromotor development from 5 to 18 years. Part 2: associated movements. Dev Med Child Neurol. (2001) 43:444–53. 10.1111/j.1469-8749.2001.tb00740.x 11463174

[18] de Kieviet JF Zoetebier L van Elburg RM Vermeulen RJ Oosterlaan J. Brain development of very preterm and very low-birthweight children in childhood and adolescence: a meta-analysis. Dev Med Child Neurol. (2012) 54:313–23. 10.1111/j.1469-8749.2011.04216.x 22283622

[19] Nosarti C Giouroukou E Healy E Rifkin L Walshe M Reichenberg A. (2008).Grey and white matter distribution in very preterm adolescents mediates neurodevelopmental outcome. Brain. 131(Pt 1):205–17. 10.1093/brain/awm282 18056158

[20] Nosarti C Nam KW Walshe M Murray RM Cuddy M Rifkin L. Preterm birth and structural brain alterations in early adulthood. Neuroimage Clin. (2014) 6:180–91. 10.1016/j.nicl.2014.08.005 25379430 PMC4215396

[21] Meng C Bauml JG Daamen M Jaekel J Neitzel J Scheef L. Extensive and interrelated subcortical white and gray matter alterations in preterm-born adults. Brain Struct Funct. (2016) 221:2109–21. 10.1007/s00429-015-1032-9 25820473

[22] Li K Sun Z Han Y Gao L Yuan L Zeng D. Fractional anisotropy alterations in individuals born preterm: a diffusion tensor imaging meta-analysis. Dev Med Child Neurol. (2015) 57:328–38. 10.1111/dmcn.12618 25358534

[23] Groeschel S Tournier JD Northam GB Baldeweg T Wyatt J Vollmer B. Identification and interpretation of microstructural abnormalities in motor pathways in adolescents born preterm. NeuroImage. (2014) 87:209–19. 10.1016/j.neuroimage.2013.10.034 24185027

[24] Gasser T Rousson V Caflisch J Jenni OG. Development of motor speed and associated movements from 5 to 18 years. Dev Med Child Neurol. (2010) 52:256–63. 10.1111/j.1469-8749.2009.03391.x 19583738

[25] Gasser T Rousson V Caflisch J Largo R. Quantitative reference curves for associated movements in children and adolescents. Dev Med Child Neurol. (2007) 49:608–14. 10.1111/j.1469-8749.2007.00608.x 17635207

[26] Rousson V Gasser T Caflisch J Largo R. Reliability of the Zurich neuromotor assessment. Clin Neuropsychol. (2008) 22:60–72. 10.1080/13854040601076702 18247219

[27] Seitz J Jenni OG Molinari L Caflisch J Largo RH Latal Hajnal B. Correlations between motor performance and cognitive functions in children born < 1250 g at school age. Neuropediatrics. (2006) 37:6–12. 10.1055/s-2006-923840 16541362

[28] Tournier JD Calamante F Connelly A. Robust determination of the ifibre orientation distribution in diffusion MRI: non-negativity constrained super-resolved spherical econvolution. Neuroimage. (2007) 35:1459–72. 10.1016/j.neuroimage.2007.02.016 17379540

[29] Tournier JD Calamante F Connelly A MRtrix: diffusion tractography in crossing fiber regions. Int J Imaging Syst Technol. (2012) 22:53–66. 10.1002/ima.22005

[30] Jones DK Cercignani M. Twenty-five pitfalls in the analysis of diffusion MRI data. NMR Biomed. (2010) 23:803–20. 10.1002/nbm.1543 20886566

[31] Tournier JD Mori S Leemans A. Diffusion tensor imaging and beyond. Magn Reson Med. (2011) 65:1532–56. 10.1002/mrm.22924 21469191 PMC3366862

[32] Bos AF Van Braeckel KN Hitzert MM Tanis JC Roze E. Development of fine motor skills in preterm infants. Dev Med Child Neurol. (2013) 55(Suppl. 4):1–4. 10.1111/dmcn.12297 24237270

[33] de Kieviet JF Piek JP Aarnoudse-Moens CS Oosterlaan J. Motor development in very preterm and very low-birth-weight children from birth to adolescence: a meta-analysis. JAMA. (2009) 302:2235–42. 10.1001/jama.2009.1708 19934425

[34] Sripada K Lohaugen GC Eikenes L Bjorlykke KM Haberg AK Skranes J. Visual-motor deficits relate to altered gray and white matter in young adults born preterm with very low birth weight. NeuroImage. (2015) 109:493–504. 10.1016/j.neuroimage.2015.01.019 25592994

[35] Murakami A Morimoto M Yamada K Kizu O Nishimura A Nishimura T. Fiber-tracking techniques can predict the degree of neurologic impairment for periventricular leukomalacia. Pediatrics. (2008) 122:500–6. 10.1542/peds.2007-2816 18762518

[36] Hoon AH Jr Stashinko EE Nagae LM Lin DD Keller J Bastian A. Sensory and motor deficits in children with cerebral palsy born preterm correlate with diffusion tensor imaging abnormalities in thalamocortical pathways. Dev Med Child Neurol. (2009) 51:697–704. 10.1111/j.1469-8749.2009.03306.x 19416315 PMC2908264

[37] Holmström L Lennartsson F Eliasson AC Flodmark O Clark C Tedroff K. Diffusion MRI in corticofugal fibers correlates with hand function in unilateral cerebral palsy. Neurology. (2011) 77:775–83. 10.1212/WNL.0b013e31822b0040 21832221

[38] Kelly CE Chan L Burnett AC Lee KJ Connelly A Anderson PJ Brain structural and microstructural alterations associated with cerebral palsy and motor impairments in adolescents born extremely preterm and/or extremely low birthweight. Dev Med Child Neurol. (2015) 57:1168–75. 10.1111/dmcn.12854 26195287

[39] Skranes J Vangberg TR Kulseng S Indredavik MS Evensen KA Martinussen M. Clinical findings and white matter abnormalities seen on diffusion tensor imaging in adolescents with very low birth weight. Brain. (2007) 130(Pt 3):654–66. 10.1093/brain/awm001 17347255

[40] Hollund IMH Olsen A Skranes J Brubakk AM Håberg AK Eikenes L. White matter alterations and their associations with motor function in young adults born preterm with very low birth weight. Neuroimage Clin. (2017) 17:241–250. 10.1016/j.nicl.2017.10.006 29159041 PMC5683190

[41] Gooijers J Swinnen SP. Interactions between brain structure and behavior: the corpus callosum and bimanual coordination. Neurosci Biobehav Rev. (2014) 43:1–19. 10.1016/j.neubiorev.2014.03.008 24661987

[42] Wahl M Lauterbach-Soon B Hattingen E Jung P Singer O Volz S. Human motor corpus callosum: topography, somatotopy, and link between microstructure and function. J Neurosci. (2007) 27:12132–8. 10.1523/JNEUROSCI.2320-07.2007 17989279 PMC6673264

[43] Grefkes C Eickhoff SB Nowak DA Dafotakis M Fink GR. Dynamic intra- and interhemispheric interactions during unilateral and bilateral hand movements assessed with fMRI and DCM. NeuroImage. (2008) 41:1382–94. 10.1016/j.neuroimage.2008.03.048 18486490

[44] van der Knaap LJ van der Ham IJ. How does the corpus callosum mediate interhemispheric transfer? A review. Behav Brain Res. (2011) 223:211–21. 10.1016/j.bbr.2011.04.018 21530590

[45] Groeschel S Hertz-Pannier L Delion M Loustau S Husson B Kossorotoff M. Association of transcallosal motor fibres with function of both hands after unilateral neonatal arterial ischemic stroke. Dev Med Child Neurol. (2017) 59:1042–1048. 10.1111/dmcn.13517 28815625

[46] Hofer S Frahm J. Topography of the human corpus callosum revisited–comprehensive fiber tractography using diffusion tensor magnetic resonance imaging. Neuroimage. (2006) 32:989–94. 10.1016/j.neuroimage.2006.05.044 16854598

[47] Takeuchi N Oouchida Y Izumi S. Motor control and neural plasticity through interhemispheric interactions. Neural Plasticity. (2012) 2012:823285. 10.1155/2012/823285 23326685 PMC3541646

[48] Adamo DE Martin BJ Brown SH. Age-related differences in upper limb proprioceptive acuity. Percept Motor Skills. (2007) 104(3 Pt 2):1297–309. 10.2466/pms.104.4.1297-1309 17879664

[49] Hoy KE Fitzgerald PB Bradshaw JL Armatas CA Georgiou-Karistianis N. Investigating the cortical origins of motor overflow. Brain Res Brain Res Rev. (2004) 46:315–27. 10.1016/j.brainresrev.2004.07.013 15571773

[50] Friston K. Sample size and the fallacies of classical inference. Neuroimage. (2013) 81:503–4. 10.1016/j.neuroimage.2013.02.057 23583356

[51] Raffelt D Tournier JD Rose S Ridgway GR Henderson R Crozier S. Apparent Fibre Density: a novel measure for the analysis of diffusion-weighted magnetic resonance images. Neuroimage. (2012) 59:3976–94. 10.1016/j.neuroimage.2011.10.045 22036682

