# Corrigendum: Motor Abilities in Adolescents Born Preterm Are Associated With Microstructure of the Corpus Callosum

**DOI:** 10.3389/fneur.2020.00162

**Published:** 2020-03-13

**Authors:** Samuel Groeschel, Linda Holmström, Gemma Northam, J-Donald Tournier, Torsten Baldeweg, Beatrice Latal, Jon Caflisch, Brigitte Vollmer

**Affiliations:** ^1^Department of Child Neurology, Children's Hospital, University of Tübingen, Tübingen, Germany; ^2^Neuropaediatric Research Unit, Department of Women's and Children's Health, Karolinska Institutet Stockholm, Stockholm, Sweden; ^3^Developmental Neurosciences Programme, UCL Institute of Child Health, London, United Kingdom; ^4^Division of Imaging Sciences and Biomedical Engineering, Department of Biomedical Engineering, Centre for the Developing Brain, King's College London, London, United Kingdom; ^5^Child Development Center and Children's Research Centre, University Children's Hospital Zürich, Zurich, Switzerland; ^6^Clinical Neurosciences, Clinical and Experimental Sciences, Faculty of Medicine, University of Southampton, Southampton, United Kingdom

**Keywords:** preterm birth, brain injury, white matter microstructure, motor abilities, diffusion magnetic resonance imaging, tractography, corpus callosum

In the original article, we neglected to include the funder **NIHR GOSH BRC**. The corrected Funding Statement appears below.

## Funding

This work was supported by an Action Medical Research project grant (SN4051). Financial support for LH was provided through the regional agreement on medical training and clinical research (ALF) between Stockholm City Council and Karolinska Institute. TB was supported by the NIHR GOSH BRC.

The authors apologize for this error and state that this does not change the scientific conclusions of the article in any way. The original article has been updated.

